# Increased Risk of Dementia in Patients with Antidepressants: A Meta-Analysis of Observational Studies

**DOI:** 10.1155/2018/5315098

**Published:** 2018-07-10

**Authors:** Yao-Chin Wang, Po-An Tai, Tahmina Nasrin Poly, Md Mohaimenul Islam, Hsuan-Chia Yang, Chieh-Chen Wu, Yu-Chuan (Jack) Li

**Affiliations:** ^1^Graduate Institute of Biomedical Informatics, College of Medical Science and Technology, Taipei Medical University, Taipei, Taiwan; ^2^Department of Emergency, Min-Sheng General Hospital, Taoyuan City, Taiwan; ^3^International Center for Health Information Technology (ICHIT), Taipei Medical University, Taipei, Taiwan; ^4^Department of Surgery, School of Medicine, Buddhist Tzu Chi University, Hualien, Taiwan; ^5^Department of Dermatology, Wan Fang Hospital, Taipei, Taiwan; ^6^TMU Research Center of Cancer Translational Medicine, Taipei, Taiwan

## Abstract

Antidepressants are the most commonly and widely used medication for its effectiveness in the treatment of anxiety and depression. A few epidemiological studies have documented that antidepressant is associated with increased risk of dementia so far. Here, our aim is to assess the association between antidepressant use and risk of dementia in elderly patients. We searched articles through MEDLINE, EMBASE, Google, and Google Scholar from inception to December 1, 2017, that reported on the association between antidepressant use and dementia risk. Data were collected from each study independently, and study duplication was checked by at least three senior researchers based on a standardized protocol. Summary relative risk (RR) with 95% CI was calculated by using a random-effects model. We selected 9 out of 754 unique abstracts for full-text review using our predetermined selection criteria, and 5 out of these 9 studies, comprising 53,955 participants, met all of our inclusion criteria. The overall pooled RR of dementia was 1.75 (95% CI: 1.033–2.964) for SSRIs whereas the overall pooled RR of dementia was 2.131 (95% CI: 1.427–3.184) for tricyclic use. Also, MAOIs showed a high rate of increase with significant heterogeneity. Our findings indicate that antidepressant use is significantly associated with an increased risk of developing dementia. Therefore, we suggest physicians to carefully prescribe antidepressants, especially in elder patients. Additionally, treatment should be stopped if any symptoms related to dementia are to be noticed.

## 1. Introduction

Dementia is a neurocognitive disorder characterized by cognitive impairment. As the life expectancy of people has been increasing, and the prevalence of dementia has been increasing dramatically [1], dementia, therefore, has appeared as a major cause of disability and dependence among older people which is triggering huge economic burdens around the world. In 2012, nearly 35.5 million of people had dementia, and this number is expected to be doubled by 2030 and approximately 115 million by 2050 [2, 3]. A few epidemiological studies have found the possible link between late-life depression and consistently an increased risk of dementia so far. These studies also mentioned that several factors like stroke, diabetes mellitus, hypertension, head trauma, and hyperlipidemia might be associated with dementia [4, 5].

Antidepressants are a commonly and widely used medication for its effectiveness in the treatment of anxiety and depression [6]. Despite their benefits in depression, concerns have emerged on their safety and the subsequent risk for dementia, but the results are still inconsistent. Previous studies have demonstrated that antidepressant drugs are responsible for adaptive process disruption. It is basically triggered by serotonin, reduces brain metabolism, and escalates the clinical conversion to dementia risk [7, 8]. Contrary, Schmitt et al. reported that treatment with an antidepressant like SSRIs may help to improve cognitive function in patients with Alzheimer's dementia [9].

Until now, there is no particular meta-analysis on the association between antidepressant use and the risk of dementia among the elderly patients. We therefore conducted a meta-analysis of observational studies to evaluate the relationship between antidepressant use and dementia risk. Finding from our present meta-analysis would assist healthcare providers to improve treatment outcome and improve the existing knowledge about antidepressant therapy. In this meta-analysis, we only included studies that reported patients having exposure to antidepressants for at least one month and included patients' mean age more than 50 years.

## 2. Methods

This meta-analysis was conducted in accordance with the Preferred Reporting Items for Systematic Reviews and Meta-analyses (PRISMA) (Supplementary Table S1) [10]. We have used similar methods in our previous published three literatures [1, 11, 12].

### 2.1. Database

In the initial stage, we developed in this study a search strategy by consulting with our senior researchers at the International Center for Health Information Technology lab. Our three senior pharmacists (MMI, TNP, and H-CY) are experts in systematic review and meta-analysis regarding drug-disease and disease-disease associations. We searched for articles on the electronic databases such as PubMed, EMBASE, and Scopus until December 1, 2017, which reported on the possible association between antidepressant use and dementia risk. We used the following search terms as medical subject headlines and keywords: (“Anti-depressant drugs” OR “SSRIs” OR “Tricyclics (TCAs)” OR “Monoamine oxidase inhibitors (MAOIs”) And (“Dementia risk”) (Supplementary Table S2). To find any missing article in our initial search, we conducted additional searches in the reference lists of all included full text. However, Google Scholar was used to finding academic articles citing eligible articles. After assembling all potential articles, we then used EndNote X7 (Thomson Reuters) to manage and remove the duplicates.

### 2.2. Eligibility Criteria

Firstly, our authors independently screened all titles and abstracts of all retrieved articles. At the initial stage, they considered all studies published only in English. Additionally, observational study designs such as case-control, cohort, and randomized control trials that reported on antidepressant use and the risk of dementia were considered in our primary search strategy. The following criteria were considered to exclude studies if (1) they published as a letter to the editor, editorial, case study, and short communications; (2) patients had less than 30 days' antidepressant use; and (3) studies had a less than one-year follow-up period.

### 2.3. Inclusion and Exclusion Criteria

In this stage, two authors (MMI and TNP) retrieved full-text articles and checked the duplication of included studies. We considered only those studies if they met the following criteria:

1. were published in English
2. reported an association between antidepressants and dementia risk
3. having at least 50 participants in both treatment and control groups
4. having results demonstrated on OR/HR with 95% CI.
5. having a more than one-year follow-up period
6. identified dementia patients with ICD-9/biochemical test or other standard procedure

However, they excluded those articles if they were not an observational study and if the participants are not patients with dementia due to antidepressants. If the study met all inclusion criteria at this stage, our two authors (MMI and TNP) further reviewed to ensure the quality of the final analysis. Any disagreements regarding quantitative information (number of participant, dementia identification, duration of therapy, etc.) between two authors (MMI and TNP) were resolved by our main supervisor (YCL) of this study. They also kept some studies for reference, if they provide valuable information regarding their associations.

### 2.4. Data Extraction and Risk Bias Assessment

We finally included four studies, and those two authors then garnered information from each of these four studies. They collected the following information from four included studies: (a) author name and publication years; (b) study duration, study location, and study design; (c) condition information (i.e., data source, condition definition, and the total number of participants); and (d) odds ratios/hazard ratios with 95% CI for summary calculation.

### 2.5. Methodological Quality Assessment

The Newcastle-Ottawa Scale (NOS) was used to evaluate the methodological quality of included observational studies (Supplementary Table S3) [13]. We evaluated included studies in three categories: selection (4 stars) and comparability (2 stars) of study groups and assessment of the outcome of interest (3 stars). The star rating system was used to indicate the quality, with 0–6 stars defined as low-quality and 7–9 stars as high-quality.

### 2.6. Statistical Analysis

In the final analysis, we calculated risk ratios (RRs) with 95% CIs to assess the risk of dementia with antidepressants. Odds ratios are close approximations of RR. We therefore combined odds ratios with HRs, resulting in a common estimate of RR [14]. A risk rate greater than 1 indicates an increased risk of dementia, and a risk rate smaller than 1 indicates a decreased risk of dementia. Statistical significance was evaluated using the 95% CIs. If the 95% CI did not include the neural value 1, we consider the risk statistically significant. In our study, we calculated the risk ratio (RR) from both case-control and cohort studies and used the most adjusted estimate available in each study due to estimation of a valid result.

A random-effects model was used to reduce the heterogeneity among the studies. We used comprehensive meta-analysis package (Version 3) to draw forest plots and subgroup estimation. The meta-analysis of proportion uses the binominal distribution for analysis. We quantified heterogeneity of the studies using the *I* ^2^ statistic, and its significance was determined based on the accompanying *P* value in Cochran's *Q* test. If the *I* ^2^ value is 0%, it indicates no heterogeneity, and increasing values represent greater amounts of heterogeneity. The *I* ^2^ values of 25%, 50%, and 75% were considered as low, moderate, and high levels of heterogeneity. However, *τ* ^2^ values arising from the random-effects models were also used to quantify heterogeneity.

## 3. Results

### 3.1. Literature Search

**Figure 1:**
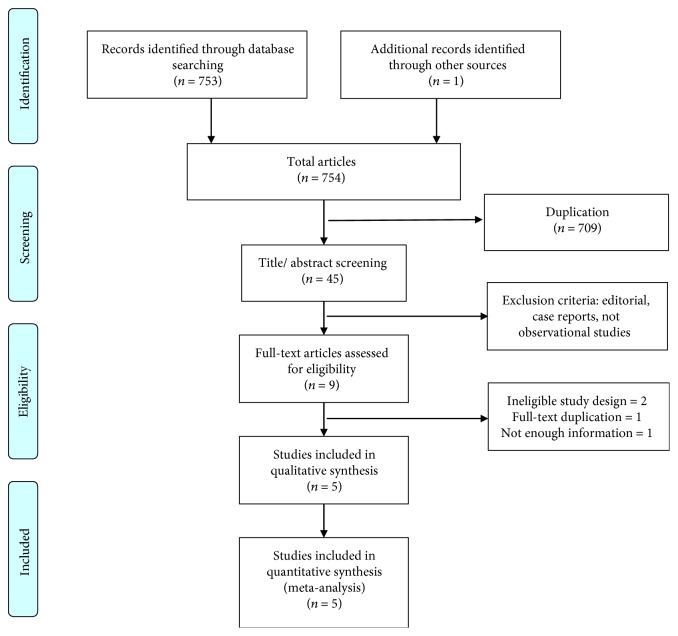
Flow chart of study selection. Diagram of study selection, adapted from the PRISMA group 2009 flow diagram.

A total of 754 records were identified through our initial database search. Of those, 745 studies were excluded based on our predetermined inclusion and exclusion criteria. We described full details in method parts. We reassessed the full text of the remaining 9 articles. Finally, 5 studies met all our inclusion criteria and were included in the final meta-analysis. A flow chart showing the study selection is presented in Figure 1.

### 3.2. Study Characteristics

The characteristics of the selected studies are presented in Table 1. The five included observational studies [15–19] were published between 2012 [19] and 2017 [18]. Among these studies, 3 were from Asia and 2 were from North America. Three studies were cohort studies [17–19], and two studies were case-control study designs [15, 16]. Two studies reported that the possible onset age of dementia was over 60 years [17, 19], and three studies reported an age of development of dementia which was below 60 years [15, 16, 18]. Almost every study mentioned antidepressant exposure, and they also evaluated the various classes of antidepressant use and dementia risk. A total of 53,955 study participants were included in our quantitative synthesis of which female patients were higher than male patients. The length of follow-up ranged from 3 to 11 years. All dementia patients were confirmed by checking the medical history, and all of the studies adjusted their results of potential confounding factors.

### 3.3. Methodological Quality of Included Studies

The Newcastle-Ottawa Scale (NOS) was used to assessed the methodological quality of included observational studies. The mean value for the five studies was 7.8 (Table 2).

### 3.4. Meta-Analysis

**Figure 2:**
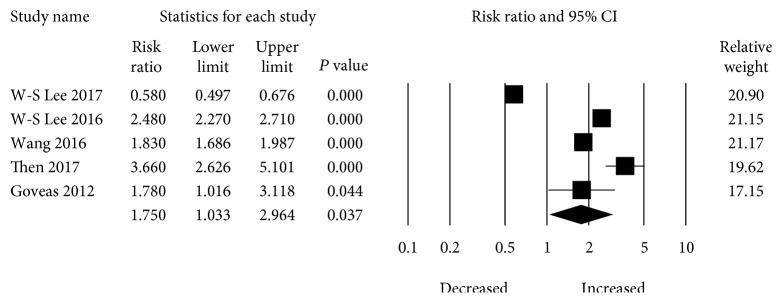
Forest plot of studies examining the association between SSRI use and dementia risk.

In the main analysis, a total of five studies evaluated the risk between selective serotonin reuptake inhibitor (SSRI) use and development of dementia. SSRI use is significantly associated with an increased risk of dementia when compared with nonuse. The overall pooled increase of dementia in patients with SSRI use was RR 1.75 (95% CI: 1.033–2.964) with significant heterogeneity present (*I* ^2^ = 98.553, *tau* ^2^ = 0.34) (Figure 2).

**Figure 3:**
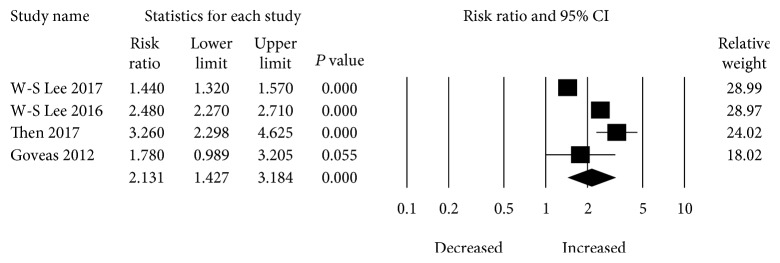
Forest plot of studies examining the association between TCA use and dementia risk.

Four studies provided the risk estimation of tricyclic (TCA) use and dementia risk. The pooled RR for dementia risk was 2.131 (95% CI: 1.427–3.184) with the patients with TCAs (Figure 3). There was also significant heterogeneity (*I* ^2^ = 96.393, tau^2^ = 0.378) among studies.

**Figure 4:**
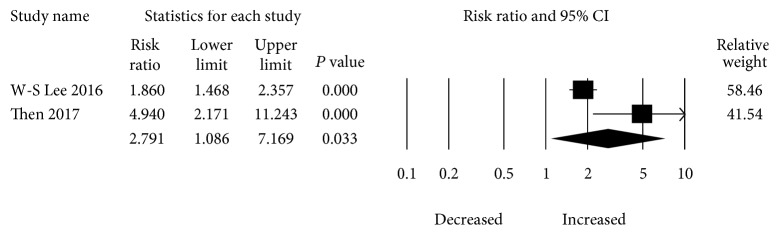
Forest plot of studies examining the association between MAOI use and dementia risk.

Two studies evaluated the impact of monoamine oxidase inhibitor (MAOI) therapy and risk of dementia. The overall pooled increase of dementia in patients with MAOI use was RR 2.791 (95% CI: 1.086–7.169) with significant heterogeneity present (*I* ^2^ = 80.019, tau^2^ = 0.382) (Figure 4).

### 3.5. Sensitivity Analysis

Lee et al. [15] evaluated the risk of dementia according to cumulative dose of individual antidepressants. Patients with SSRIs had higher risk of dementia [(HR = 2.04, 95% CI: 1.80–2.31; HR = 2.10, 95% CI: 1.85–2.39; HR = 2.96, 95% CI: 2.60–3.37; and HR = 3.07, 95% CI: 2.69–3.51)] for <840 mg, 841–3000 mg, 3001–10,500 mg, and >10,500 mg. Contrarily, tricyclic was associated with a decreased risk of dementia [(HR = 0.44, 95% CI: 0.38–0.51, HR = 0.26, 95% CI: 0.22–0.30; HR = 0.20, 95% CI: 0.17–0.24; and HR = 0.13, 95% CI: 0.11–0.15)] for <840 mg, 841–3000 mg, 3001–10,500 mg, and >10,500 mg, respectively. Then et al. [18] investigated gender, different age groups, and dementia risk. Males had a higher risk of developing dementia (HR = 3.59, 95% CI: 2.64–4.88) than female patients (HR = 4.45, 95% CI: 3.11–6.37). Additionally, patients aged 45–65 had a higher risk (HR = 8.34, 95% CI: 4.45–15.61) than patients aged ≥65 years (HR = 3.84, 95% CI: 2.98–4.94). Goveas et al. [19] provided information about developing adverse effects such as mild cognitive impairment (HR = 1.70, 95% CI: 1.14–2.54) and probable dementia (HR = 1.24, 95% CI: 0.71–2.17).

### 3.6. Publication Bias

**Figure 5:**
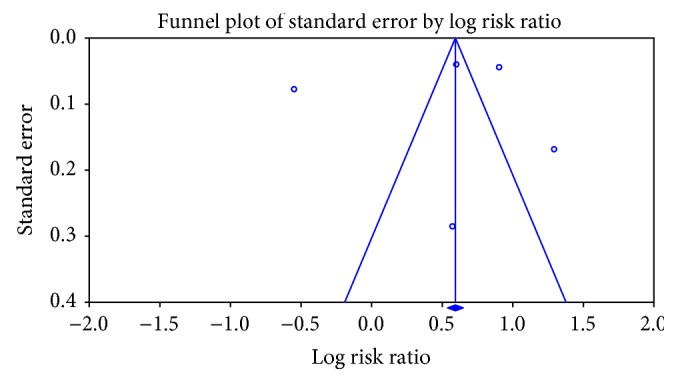
Funnel plot showing the association between antidepressant use and dementia risk.

In our study, we showed the visual interpretation and test for asymmetry of the funnel plot of the publications. However, it is well established that the test of asymmetry will not be reliable when the included study number is not substantial [20].  Figure 5 depicts the funnel plot, indicating the presence of publication bias. Egger's regression test was used to present the funnel asymmetry, and it showed a highly significant publication bias (*P* value = 0.79).

## 4. Discussion

### 4.1. Main Outcome

This current meta-analysis of observational epidemiological studies suggests that use of antidepressants increased a risk of developing dementia among the elderly population when compared with nonusers. Patients with monoamine oxidase inhibitor therapy had a higher risk of developing dementia than those with tricyclic and selective serotonin reuptake inhibitor therapy. Of importance, these findings would make physicians more cautious when they will consider an antidepressant treatment to their patients with depression. However, the decision to prescribe antidepressant therapy should be made in the light of the stringent clinical evaluation of benefits and risks, more particularly when physicians will prescribe antidepressants to a patient with depression. If antidepressant therapy is needed, it should be used judiciously and for the shorter period of time. Additionally, healthcare providers should routinely monitor the symptom of dementia risk factors such as memory loss, confusion, and disorientation while initiating or rewriting antidepressants to patients.

### 4.2. Biological Evidences

The biological mechanisms of the association between antidepressants and risk of developing dementia are still unclear. However, there are various possible ways of explanations for their association. First, the onset of depression before 65 years' age might be linked to the development of dementia and more recent depressive symptoms might link to the prodromal phase of dementia [7, 21]. Several biological studies reported that apolipoprotein (APOE) *ɛ*4 is a major known genetic risk factor that synergistically interacts with depression. Their interaction enhances the possibility of dementia risk, but it is not still well established [22, 23]. Second, use of antidepressants might prompt the imbalance of various neurobiological pathways. Therefore, it could help to elevate oxidative and nitrosamine stress as well as inflammation. Additionally, it might promote mitochondrial dysfunction, increased apoptosis, and finally diminished neurotrophic support [24]. Third, Steffens et al. reported that antidepressants increase subclinical cerebrovascular disease risk and promote cognitive decline by elevating potential oxidative stress [25]. Furthermore, to confirm the possible biological mechanism/link, more experimental and biological models are warranted.

### 4.3. Strengths and Limitations

Our study has several strengths. First, there is an enhanced statistical power to evaluate any association between antidepressant use and dementia risk. Second, this study evaluates the association in great detail and showed an association of different categories of antidepressants. However, our study has several limitations that need to be addressed. First, we included a small number of studies in our analysis. Second, a meta-analysis of epidemiological studies always has some unmeasured or uncontrolled confounding factors from the original studies. Our study findings had some degree of heterogeneity even though we used a random-effects model in our study analysis that helps to reduce publication bias. Third, we did not provide any information regarding dose differential and risk of dementia due to an insufficient amount of information. Fourth, duration of antidepressant and dementia risk were not evaluated because of restrained data. Fifth, types of dementia risk with antidepressant use were not investigated due to lack of information. Sixth, females are more prevalent to depression and anxiety than males are, and females act differently in the metabolism and distribution of antidepressants [26, 27], but we could not categorize dementia risk on the basis of gender. Finally, we were unable to include randomized controlled trials due to the absence of data.

### 4.4. Recommendation

Till now, no particular treatments are available to improve cognitive and behavioral symptoms of dementia patients. Additionally, antidepressant prescription has been increasing and concerns have been increased due to antidepressant therapy. Therefore, treatment of depression patients with antidepressant therapy should be assessed on a timely basis to reduce any unfavorable effects. If any symptoms are observed, then physicians need to stop antidepressant therapy or treat patients with a low dosage or shorter period of time. It is also indispensable to inform patients or their family about the inauspicious consequence of antidepressant therapy and other treatment options available for their current physical condition. However, to ensure safe and effective treatment, it is high time to provide proper guidelines for patients and healthcare providers.

### 4.5. Unanswered Questions and Future Direction

Since our study summarized results from several epidemiological studies, this is why we acknowledge that findings from this study could not clarify whether the observed association between antidepressants and dementia risk is the causal effect or other unmeasured confounding variables [28]. Hence, larger long-term prospective randomized control trials and biological studies are warranted to justify a possible biological mechanism which links dementia risk or rebut their association.

## 5. Conclusion

To our knowledge, this meta-analysis shows the most rigorous and precise analysis regarding antidepressant use and the risk of dementia. Our findings showed a significantly increased risk of dementia with antidepressant therapy. Healthcare providers might preferentially avoid unnecessary prescription of antidepressants to the patients if any symptoms regarding dementia are noticed. Additionally, proper guidelines should be provided for quality and safety treatment.

## Figures and Tables

**Table 1:** Characteristics of five included studies regarding antidepressants use and dementia risk.

Author/year	Country	Study duration	Study design	Adjustments	Results
Wei-Sheng Lee-2016 [15]	Taiwan	2005–2011	Case-control	1, 2, 3, 4, 5, 6, 7, 8, 9	For SSRIs, OR = 2.48 (95% CI: 2.27–2.71) For MAOIs, OR = 1.86 (95% CI: 1.47–2.36) For TCAs, OR = 1.44 (95% CI: 1.32–1.57),
Wei-Sheng Lee-2017 [16]	Taiwan	2005–2011	Case-control	1, 2, 3, 4, 5, 6, 7, 8, 9	For SSRIs, OR = 0.58 (95% CI: 0.50–0.69) For TCAs, OR = 1.02 (95% CI: 0.89, 1.17 For NGAs, OR = 4.23 (95% CI: 3.34, 5.37)
Wang 2016 [17]	USA	1991–2010	Cohort	1, 2, 3, 6, 9, 10 , 11, 12, 13, 14, 15, 16, 17, 18	For SSRIs, HR = 1.83 (95% CI) For non-SSRIs, HR = 1.50 (95% CI)
Then 2017 [18]	Taiwan	2003–2006	Cohort	1, 2, 3, 4, 8, 9, 19, 20	For SSRIs, HR = 3.66 (95% CI: 2.62–5.09) For SNRI, HR = 4.73 (95% CI: 2.54–8.80) For TCAs, HR = 3.26 (95% CI: 2.30–4.63) For MAOIs, HR = 4.94 (95% CI: 2.17–11.24)
Goveas 2012 [19]	USA	1996–2007	Cohort	1, 2, 3, 4, 6, 9, 10, 11, 21	For SSRIs, HR = 1.78 (95% CI: 1.01–3.10) For TCAs, HR = 1.78 (95% CI: 0.99–3.21).

(1) age, (2) gender, (3) diabetes, (4) hypertension, (5) stroke, (6) coronary artery disease, (7) head injury, (8) anxiety, (9) depression, (10) smoking, (11) body mass index, (12) cancer, (13) COPD, (14) liver disease, (15) hyperlipidemia, (16) renal disease, (17) thyroid disease, (18) cerebrovascular disease, (19) insomnia, (20) CCI, and (21) history of alcohol consumption.

**(a):** 

Case-control study	Selection				Comparability	Exposure			Total
	Definition adequate	Representativeness of the cases	Selection of controls	Definition of controls	Control for important factor or additional factor	Ascertainment of exposure	Same method of ascertainment for cases and controls	Nonresponse rate	(0–9)
Wei-Sheng Lee-2017 [16]	^∗^	^∗^	^∗^	^∗^	^∗∗^	^∗^	^∗^		8
Wei-Sheng Lee-2016 [17]	^∗^	^∗^	^∗^	^∗^	^∗∗^	^∗^	^∗^		9

Note: a “star (^∗^)” system of the Newcastle-Ottawa Scale (NOS) has been developed for the methodological quality assessment: each study can be awarded a maximum of one star for each numbered item within the selection and exposure categories, while a maximum of two stars can be given for the comparability category.

**(b):** 

Cohort study	Selection				Comparability	Exposure			Total
	Selection of nonexposed cohort	Representativeness of the cohort	Ascertainment of exposure	Outcome of interest	Comparability of cohorts on the basis of the design or analysis	Assessment of outcome	Follow-up long enough for outcomes to occur	Adequacy of follow-up of cohorts	(0–9)
Wang 2016 [17]	^∗^	^∗^	^∗^	^∗^	^∗^	^∗^	^∗^		7
Then 2017 [18]	^∗^	^∗^	^∗^	^∗^	^∗∗^	^∗^	^∗^	^∗^	9
Goveas 2012 [19]	^∗^	^∗^		^∗^	^∗^	^∗^	^∗^		6

Note: a “star (^∗^)” system of the Newcastle-Ottawa Scale (NOS) has been developed for the methodological quality assessment: each study can be awarded a maximum of one star for each numbered item within the selection and exposure categories, while a maximum of two stars can be given for the comparability category.

## Data Availability

The data used to support the findings of this study are available from the corresponding author upon request.

## Supplementary Materials

Supplementary Materials Table S1: PRISMA 2009 checklist. Table S2: search strategy. Table S3: modified Newcastle-Ottawa Scale for risk of bias assessment.
